# Changes in physical and technical match performance variables in football players promoted from the Spanish Second Division to the First Division

**DOI:** 10.5114/biolsport.2024.127386

**Published:** 2023-08-08

**Authors:** Jordi Ferrandis, Juan Del Coso, Víctor Moreno-Pérez, Roberto López-Del Campo, Ricardo Resta, Joaquín González-Rodenas

**Affiliations:** 1 Sport Sciences Research Centre, Rey Juan Carlos University, Fuenlabrada, Madrid, Spain; 2 Catholic University of Valencia, “San Vicente Mártir”, Valencia, Spain; 3 Sports Research Center, Miguel Hernandez University of Elche, Alicante, Spain; 4 Department of competitions and Mediacoach, LaLiga, Madrid, Spain

**Keywords:** Soccer, Match analysis, Elite athletes, Team sports, Match demands

## Abstract

The aim of this study was to compare physical and technical match performance variables in football players who competed in the Spanish second division for one season and were promoted to the top (first) division in the following season. A total of 97 male outfield football players who were promoted from the second to the first division of the Spanish professional football league within the same team were analysed. Data were recorded using the TRACAB (ChyronHego, New York, USA) multicamera computerised optical tracking system during five seasons (2015–2016 to 2019–2020). A one-way ANOVA repeated measures analysis showed that players executed a greater number of high-intensity running (HIR) efforts (P < 0.001; ES: 0.258), as well as covering greater HIR distance (P < 0.010; ES: 0.106) and total running distance (TD) (P < 0.010; ES: 0.080), when they played in the first division compared with the second division. Moreover, players performed a lower number of passes (P < 0.01; ES = 0.116), short passes (P < 0.01; ES = 0.106), long passes (P < 0.05; ES = 0.067), dribbles (P < 0.001; ES = 0.146) and shots (P < 0.01; ES = 0.074) in the first division compared to the second division. No significant differences were found for any of the defensive variables evaluated. In conclusion, being promoted from the second to the first division of professional football requires players to adapt to greater physical demands and a reduced number of technical actions.

## INTRODUCTION

Professional football players require a great capacity for performing intermittent high-intensity physical efforts interspersed with fast technical and tactical actions [1]. In the last decades, professional football has evolved and now the game demands a higher quantity of high-intensity running (HIR) efforts and greater technical accuracy [2, 3, 5, 6, 7, 8]. This physical and technical evolution may be due to the interaction of multiple factors such as the improvements in the physical preparation of players [8], the new trends in tactical periodization [4], as well as the incorporation of modern technologies to monitor the competition and the training process [9, 10]. All these factors contribute to implementing better control and management of professional players’ physical load within each game and throughout the season.

A great number of studies have focused on the analysis of physical and technical performance of top categories of professional football [11]. However, professional football in most national leagues encompasses two or more categories that retain the same structure of competition. In these national leagues, teams may promote/relegate between categories, which exposes some players to play in both levels. Hence, the comparison of game characteristics between the first and second categories of professional football in national leagues deserves investigation to understand the process that the players undergo when they are promoted to a higher category. This would allow a better understanding of how players’ demands can change between different competitive levels to allow faster adaptation to the new category [12, 13].

In this regard, the literature that has analysed how the competitive level affects match performance physical and technical variables in professional football is scarce and has provided controversial findings.

For instance, it was concluded [12] that English Premier League players covered less total and HIR distance compared to players playing at lower competitive levels such as the Championship and League One. Also, this study observed superior technical ability in the Premier League compared to the lower-level competitions. In the same way, it was observed [20] that players in the Championship covered more total, high-speed running and sprinting distances than those in the English Premier League.

In contrast with these results, recent reports in the Spanish *LaLiga* found that HIR distances were greater in the Spanish first division compared to the second division [13, 14] and the top-ranking teams in the first division covered significantly greater distances than the other teams in the same division and in the second division [15].

Moreover, the referred study [15] reported that first division teams performed more passes and registered a higher percentage of successful passes than second division ones. When analysing different playing positions, greater high-intensity actions were found for the central defenders (CD), wide midfielders (WM), and attackers in the first division compared to the second division [16].

However, all these investigations were cross-sectional because they analysed different teams competing in each category. From a player development perspective, there is a lack of scientific evidence on the possible changes in game demands that the same player can have when being promoted from the second division to the first division. To the best of our knowledge, only one study [17] has compared the individual physical and technical performance of the same group of players when being promoted from the English second division to the Premier League. In Morgan’s study, the distance covered over the season was slightly higher in the first division with no effect on sprint and high-intensity distances, although the limited sample of players used in this investigation may limit the extrapolation of these results to other teams or competitions.

Overall, the different methodological approaches and the contradictory findings impede understanding of how the physical and technical demands of the game change for players who are promoted to a higher category of professional football.

Therefore, the aim of this study was to compare physical and technical match performance variables of football players who were promoted from the Spanish second division to the first division within the same team. Based on previous studies in Spanish football, we hypothesized that players would perform more HIR efforts and cover more HIR distance in the first division compared to the second division, regardless of their playing position.

## MATERIALS AND METHODS

### Sample

A total of 97 professional outfield football players who played in the second division and were promoted with the same team to the first division of the Spanish professional football league were included in this investigation (Table 1). To achieve this sample of players, data were gathered from five seasons (from 2015–2016 to 2019–2020) with a total of 12 teams promoted to the first division. The only inclusion criterion was the selection of players who played a minimum of four and a maximum of thirty-eight full matches in each division, maintaining his outfield position. Goal-keepers were excluded from the study because of their different match performance demands compared to outfield players. Players were classified into five playing position roles – central defenders (CDs) (n = 26), full backs (FBs) (n = 19), central midfielders (CMs) (n = 32), wide midfielders (WMs) (n = 11) and forwards (FWs) (n = 9) – following the classification of multiple previous studies [6, 7, 18].

**TABLE 1:** Description of the players evaluated in each team (N), average number of matches per player and team ranking position at the end of the season in both divisions.

Period	Team	N	2^nd^ Division		1^st^ Division	

			Matches	Ranking Position	Matches	Ranking Position
2015–2017	Team A	4	37.7 ± 0.5	1	25.5 ± 5.4	9
	Team B	6	32.2 ± 9.4	6	26.3 ± 7.6	19
	Team C	9	30.2 ± 9.3	2	24.8 ± 6.8	17

2016–2018	Team D	10	34.1 ± 4.3	1	26.5 ± 7.4	15
	Team E	9	29.2 ± 12.0	2	29.1 ± 9.6	10
	Team F	6	35.1 ± 7.0	3	27.3 ± 9.3	8

2017–2019	Team G	7	30.8 ± 8.4	1	28.2 ± 9.6	20
	Team H	8	32.2 ± 8.6	2	28.9 ± 8.1	19
	Team I	12	32.3 ± 5.4	5	27.2 ± 8.7	16

2018–2020	Team J	7	32.7 ± 9.0	2	24.3 ± 9.6	7
	Team K	9	31.7 ± 7.0	1	26.5 ± 6.9	10
	Team L	10	31.0 ± 7.0	5	29.1 ± 8.9	19

### Variables and Procedures

The design of the study is intrasubject comparative, where the mean value of the matches that the player played in the second division was compared with the mean value of the matches that the same player played in the first division in the following season.

Data were collected using the TRACAB (ChyronHego, New York, USA) multicamera computerised optical tracking system, which has a sampling frequency of 25 Hz, and processed using the Mediacoach software (*LaLiga*, Madrid, Spain). This system has been deemed as a valid and reliable tool to analyse football performance activities [19]. In accordance with the ethical guidelines of *LaLiga*, this investigation does not include information that identifies football players.

The variables of the present study were divided into two categories: individual match running performance variables and technical performance variables. For the first group of variables, the number of efforts performed at HIR (number of HIR), the distance covered at HIR (i.e., > 21 km · h^−1^, in m) and the total distance covered (TD, in m) were obtained for each match. The technical performance variables included total number of passes, passing accuracy (%), passing verticality (%) (proportion of passes with forward direction from the total number of passes), number of short passes (those of less than 30 m), number of long passes (those of more than 30 m), dribbles, shots, aerial duels, interceptions, clearances, and tackles.

### Statistical Analysis

All analyses were conducted using statistical software (SPSS Inc., V20, Chicago, IL, USA). Descriptive statistics were implemented through the mean (M) and standard deviation (± SD). Data normality was examined using the Kolmogorov-Smirnov test. One-way repeated-measures ANOVA was used to calculate the differences in match performance variables between the first and second division. Also the playing position was considered an inter-subject factor to examine its interaction with the competitive level. *Post hoc* analysis was performed using the Bonferroni test to examine the differences between the first and second division for each playing position. The magnitudes of the differences for all variables were analysed using partial eta squared (ES) (ηp^2^).

## RESULTS

**FIG. 1:**
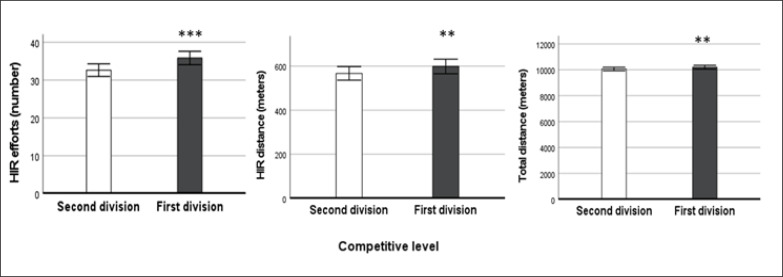
Number of high-intensity running (HIR) efforts, HIR distance and TD (total distance) covered according to the competitive level in Spanish professional football players. * = P < 0.05; ** = P < 0.01; *** = P < 0.001.

**FIG. 2:**
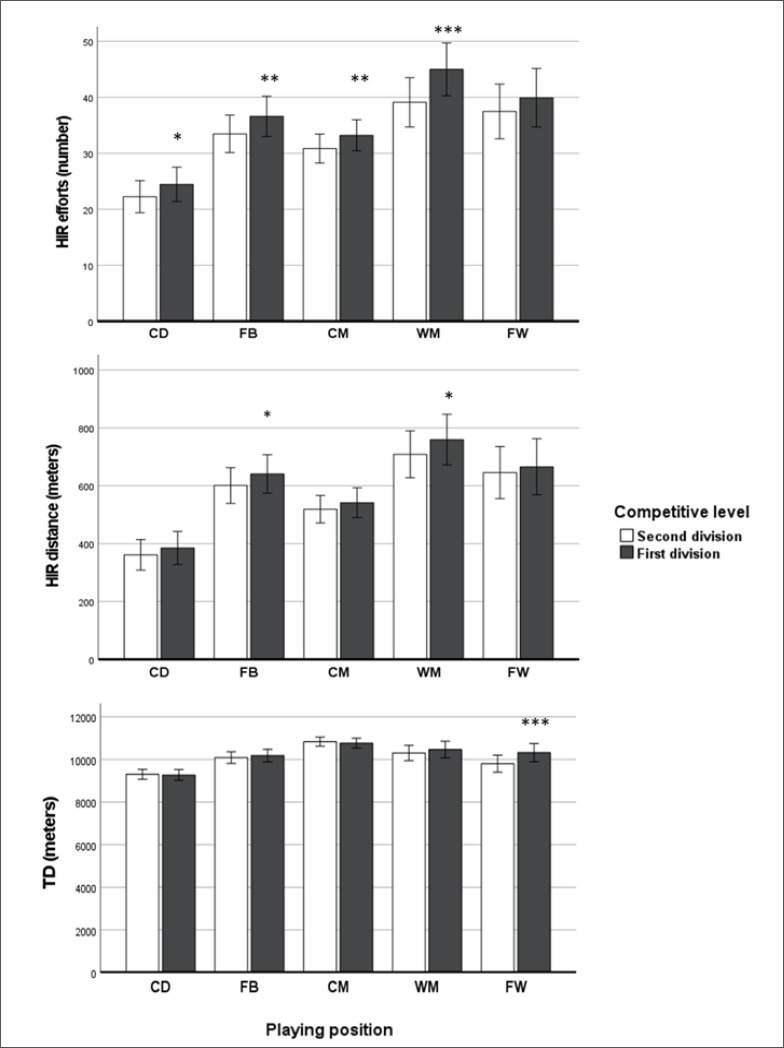
Number of high-intensity running (HIR) efforts, HIR distance covered and total distance (TD) according to playing position and competitive level in Spanish professional football players. Note: * = P < 0.05; ** = P < 0.01; *** = P < 0.001.

Figures 1 and 2 present physical performance variables of players in the first vs second division. Players executed a greater number of HIR efforts (P < .001; ES: 0.258), HIR distance (P < .01; ES: 0.106) and TD (P < .01; ES: 0.080) when they played in the first division compared with the second division (Figure 1). For TD, an interaction effect between the competitive level and the playing position was found (P < .01; ES: 0.147).

In Figure 2 it can be observed that all playing positions, except for the FWs, performed more HIR efforts when competing in the first division than in the second division. As for HIR distance, FBs (641.5 ± 140.4 vs 601.4 ± 130.1; P < 0.05) and WM (759.7 ± 117.0 vs 708.9 ± 100.1; P < 0.05) covered greater distance in the first division than in the second division, whereas no significant differences were found for the rest of playing positions. Concerning TD, no differences were found for all playing positions except for FWs, who covered greater TD in the first division compared to the second division (10320.7 ± 897.8 vs 9803.5 ± 621.7; P < 0.001).

Table 2 shows the match offensive and defensive technical variables of players according to the competitive level, as well as the interaction effect of their playing position. Repeated-measures analysis indicated that players performed fewer passes (P < 0.01; ES = 0.116), short passes (P < 0.01; ES = 0.106), long passes (P < 0.05; ES = 0.067), dribbles (P < 0.001; ES = 0.146) and shots (P < 0.01; ES = 0.074) in the first division compared to the second division. Statistical analysis showed no significant differences for passing accuracy and passing verticality between categories.

**TABLE 2:** Comparative analysis of offensive and defensive technical variables according to the competitive level and its interaction with playing position (mean ± SD).

Variable (unit)	Competitive level		Division		Division*Position			

	Second Division	First Division	F1	P2	ES^3^	F1	P2	ES^3^
Total passes (n)	42.6 ± 9.9	38.7 ± 9.1	12.13	0.001	0.116	1.50	0.209	0.061
Passing accuracy (%)	73.3 ± 6.8	73.8 ± 6.9	0.64	0.425	0.007	0.55	0.695	0.024
Passing verticality (%)	65.8 ± 11.2	65.8 ± 10.1	0.57	0.449	0.006	1.06	0.379	0.044
Short passes (n)	35.3 ± 8.5	32.2 ± 7.9	10.85	0.001	0.106	0.98	0.418	0.041
Long passes (n)	7.3 ± 3.8	6.5 ± 3.4	6.59	0.012	0.067	2.42	0.054	0.095
Dribbles (n)	2.0 ± 1.8	1.7 ± 1.6	15.67	< 0.001	0.146	1.83	0.128	0.074
Shots (n)	1.1 ± 0.9	1.0 ± 0.8	7.38	0.008	0.074	1.98	0.103	0.079
Aerial duels (n)	3.4 ± 2.0	3.2 ± 2.0	0.04	0.839	0.000	3.81	0.006	0.142
Interceptions (n)	4.5 ± 1.4	4.5 ± 1.3	1.04	0.309	0.011	1.61	0.178	0.066
Tackles (n)	0.7 ± 0.4	0.7 ± 0.4	2.01	0.159	0.021	0.33	0.853	0.014
Clearances (n)	2.5 ± 1.7	2.4 ± 1.7	0.19	0.659	0.002	0.39	0.814	0.017

Note: F^1^ = F-value P^2^ = ANOVA of repeated measures; ES^3^ = effect size: partial eta squared

In addition, no changes were found for any of the defensive variables such as aerial duels, interceptions, tackles, and clearances. No significant interaction effects were found in any technical variable, except for aerial duels (P < 0.01; ES = 0.142).

Table 3 displays the match offensive and defensive technical variables according to the competitive level categorized by playing positions. In this regard, CBs completed lower numbers of passes, short passes, long passes (P < 0.01) and aerial duels (P < 0.05) in the first division compared to the second division. With regard to FBs, players performed fewer passes, short passes, long passes and dribbles (P < 0.05) in the first division than in the second division. In the position of CMs, players performed fewer passes, short passes, long passes (P < 0.01) and aerial duels (P < 0.05) in the first division. For WMs, no significant differences were found for any of the technical variables. Finally, FWs performed fewer dribbles and shots (P < 0.01) but more aerial duels (P < 0.01) and interceptions (P < 0.05) in the first division compared to the second division.

**TABLE 3:** Comparative analysis of offensive and defensive technical variables according to the competitive level and playing positions (mean ± SD).

Variable (unit)	Competitive level			p

	Playing position	Second Division	First Division	
Total passes (n)	CD	41.4 ± 5.3	36.1 ± 4.1	0.001
	FB	48.0 ± 7.1	43.7 ± 5.7	0.014
	CM	46.1 ± 10.1	41.4 ± 10.7	0.001
	WM	39.3 ± 9.0	38.0 ± 8.6	0.565
	FW	26.5 ± 7.4	27.2 ± 9.8	0.781

Passing accuracy (%)	CD	74.6 ± 7.2	75.1 ± 6.6	0.594
	FB	72.8 ± 5.1	72.4 ± 3.9	0.679
	CM	75.7 ± 6.1	76.4 ± 6.5	0.390
	WM	70.6 ± 5.6	70.0 ± 6.8	0.690
	FW	66.1 ± 7.3	68.1 ± 9.2	0.216

Passing verticality (%)	CD	78.4 ± 5.6	76.8 ± 4.6	0.146
	FB	67.7 ± 6.8	68.0 ± 6.2	0.844
	CM	62.3 ± 8.1	62.6 ± 6.9	0.798
	WM	54.0 ± 6.2	55.3 ± 4.0	0.426
	FW	52.0 ± 5.3	54.2 ± 11.5	0.246

Short passes (n)	CD	31.8 ± 6.6	27.6 ± 4.8	0.002
	FB	38.6 ± 6.8	35.2 ± 4.3	0.031
	CM	39.5 ± 8.0	35.9 ± 8.4	0.002
	WM	34.8 ± 7.6	33.3 ± 7.1	0.444
	FW	24.5 ± 6.9	24.8 ± 9.2	0.892

Long passes (n)	CD	9.7 ± 3.0	8.4 ± 3.0	0.001
	FB	9.4 ± 2.2	8.5 ± 2.1	0.021
	CM	6.6 ± 3.7	5.5 ± 3.4	0.002
	WM	4.5 ± 1.8	4.7 ± 1.9	0.693
	FW	2.0 ± 1.5	2.4 ± 2.2	0.515

Dribbles (n)	CD	0.4 ± 0.3	0.4 ± 0.3	0.764
	FB	1.7 ± 1.2	1.3 ± 0.9	0.042
	CM	2.0 ± 1.8	1.7 ± 1.6	0.321
	WM	4.7 ± 1.3	4.2 ± 1.6	0.056
	FW	3.8 ± 2.1	2.9 ± 1.5	0.005

Shots (n)	CD	0.5 ± 0.2	0.4 ± 0.2	0.613
	FB	0.5 ± 0.3	0.6 ± 0.3	0.754
	CM	1.4 ± 0.8	1.2 ± 0.8	0.107
	WM	1.3 ± 0.7	1.2 ± 0.8	0.489
	FW	2.7 ± 1.0	2.2 ± 1.2	0.003

Aerial duels (n)	CD	4.4 ± 1.7	3.8 ± 1.3	0.021
	FB	2.4 ± 1.1	2.5 ± 1.3	0.829
	CM	3.4 ± 2.3	2.9 ± 2.1	0.036
	WM	1.9 ± 1.0	1.9 ± 1.0	0.985
	FW	4.5 ± 2.2	5.7 ± 3.2	0.008

Interceptions (n)	CD	5.1 ± 1.2	5.3 ± 1.1	0.561
	FB	4.9 ± 0.7	4.7 ± 0.8	0.369
	CM	4.6 ± 1.3	4.4 ± 1.3	0.456
	WM	3.7 ± 1.2	3.8 ± 1.1	0.780
	FW	2.0 ± 0.8	2.9 ± 1.4	0.033

Tackles (n)	CD	0.7 ± 0.2	0.6 ± 0.3	0.247
	FB	0.8 ± 0.3	0.8 ± 0.4	0.894
	CM	0.8 ± 0.5	0.7 ± 0.4	0.193
	WM	0.8 ± 0.4	0.7 ± 0.3	0.240
	FW	0.5 ± 0.3	0.5 ± 0.6	0.909

Clearances (n)	CD	4.7 ± 1.1	4.6 ± 1.3	0.517
	FB	3.1 ± 0.7	2.9 ± 0.8	0.232
	CM	1.3 ± 0.8	1.3 ± 0.7	0.758
	WM	1.1 ± 0.8	1.1 ± 0.9	0.963
	FW	0.9 ± 0.5	1.1 ± 0.6	0.564

## DISCUSSION

The aim of this study was to compare the individual match physical and technical performance of football players who were promoted from the Spanish second division to the first division within the same team. The main finding of this study is that football players covered more HIR distance and performed more HIR efforts when playing in the first division compared to the second division. Also, regarding the technical side, players in the first division executed a lower number of offensive technical variables than in the second division, including lower frequency of passing, dribbling and shooting, while no significant differences between divisions were found for the defensive variables.

These results are in line with previous studies in Spanish football, which found that the HIR distance and the number of sprints were significantly higher in first division teams than in second division teams [13, 14]. In this respect, our study is innovative because it followed a longitudinal analysis by analysing changes within the same players, and now we can confirm that is the player who adapt to the demands of the category beyond simple variation between the teams competing in each category. In contrast, our findings are contrary to those observed in previous studies performed in English football, which revealed that players playing in the second division covered more HIR distance [12] and TD [20] than those playing in the first division.

In the same way, our findings differ from those of other researchers [17] who found no differences in sprint performance and HIR distance between the first and second division in English football after promotion. This lack of agreement between studies may be due to differences in the different tactical and physical contexts experienced by the players in English and Spanish leagues [21, 22].

It is important to note that the change in the physical demands of the game from the second to the first division of Spanish football was different depending on the playing position. In this sense, the present study revealed that all playing positions except FWs performed more HIR efforts. Possibly, playing in the top division should expose players to a more demanding tactical scenario with high-skill opponents than in the second division [16]. In this context, the playing tempo and technical accuracy of matches are higher, so that more HIR efforts may be necessary to adapt to the new tactical demands. Particularly, FBs and WMs were the playing positions that registered the greatest increase in HIR distance in the first division.

Multiple studies have observed that FBs and WMs are the playing positions with more HIR distance covered during matches [23, 24, 25], probably due to their wide positioning where players should run long distances both to attack and defend. In the case of FBs, it seems very important to perform HIR runs when performing recovery runs, covering space, supporting their teammates in attack, as well as when overlapping [26]. For WMs, it is more frequent to perform HIR runs by carrying the ball and by supporting the offensive play than the rest of the playing positions [26]. Thus, our findings highlight the even greater importance of covering HIR distance in FBs and WM after promotion to the first division. From a practical standpoint, the strength and conditioning staff of teams promoted to a higher division should focus on enhancing the running capacity of all the players, but especially at high intensity and for players in wide positions.

Concerning TD, although significant differences between divisions were found when all playing positions were analysed conjointly, only FWs covered significantly more distance in the first division versus the second division. This result indicates that FWs are required to perform an extra effort in the first division, probably related to the higher playing tempo of the new category, as well as the possible necessity of having more involvement and participation in the defensive phase in comparison with the second division.

Regarding technical variables, our findings indicated that players, especially CBs, FBs and CM, performed fewer passes, short passes and long passes when playing in the first division, although no differences were found in terms of passing accuracy and playing verticality. Furthermore, FBs, WMs and FWs performed a lower quantity of dribbles, and FWs registered fewer shots in the first division compared to the second division. It is interesting to observe how the significant changes are specific to the playing positions, showing the relevance of different technical demands according to the tactical role assumed per each player, as previous studies have pointed out in professional football [27]. These results contrast with several studies that revealed that players at a higher competitive level/category performed a greater quantity of technical actions than players competing at lower levels such as second division [12, 16]. In this sense, it is crucial to highlight that those previous studies compared categories independently, but our study compared the performance of the same players when competing in both categories. Interestingly, our findings suggest that the individual performance of players could be influenced by the different team status and ranking position in each division. In the second division, the sampled teams and players achieved promotion, revealing that they were the most successful teams in the league, which probably entailed high values of offensive technical indicators. When being promoted to the first division, professional football players had to adapt to a more demanding context with lower level of offensive success, which may explain the lower offensive values registered in comparison with the second division.

As for defensive variables, no general differences between divisions were found for the variables analysed. Nevertheless, the FWs were the players who experienced the biggest change in this aspect, so that they registered slightly higher values of interceptions and aerial duels in the first division than in the second division. These changes may be due to higher defensive demands when playing in the first division, which also entailed greater efforts in terms of TD covered. Otherwise, the rest of playing positions maintained a stable defensive performance in both competitive levels.

This study has limitations that require acknowledgment. First, all the variables evaluated in this research are based on match statistics, which limits the capacity to capture the complex dynamic of football tactics based on the interactions and synergies between players and teams [28]. Additionally, the study was performed with data from the Spanish football league of professional male players, and the results should not be extrapolated to other leagues, other categories or women’s football.

However, the large sample of players and the methodology implemented based on a repeated measures analysis reinforce the consistency of the findings, which have important practical applications.

### Practical Applications

This study has strong practical applications to professional football and for those teams being promoted to a higher category. Firstly, it shows how players from the Spanish second category being promoted to the first division have to face a more challenging game, especially in terms of total distance and HIR. Interestingly, the magnitude of the physical demands of the game depends on the playing position, and it may be higher in players with wider field positions. Therefore, the outcomes of this study could be useful for coaches and fitness coaches to design training strategies that allow optimal preparation of players when they achieve promotion, especially to make them aware of the greater demands in the higher league. Secondly, the offensive technical values were found to be lower in the first division than in the second division, having a strong relationship with the specific playing positions. This finding suggests that players must become adapted to the new technical demands in the new category, reducing their offensive involvement with the ball while maintaining their capacity to defend.

## CONCLUSIONS

In conclusion, male professional football players covered more HIR distance and performed more HIR efforts when playing in the first division of Spanish football, compared to the second division. In addition, players in the first division registered a lower number of offensive technical variables than in the second division, including lower frequency of total passes, short passes, long passes, dribbles, and shots, while no general significant differences between divisions were found in the defensive variables.

## Supplementary Material

Changes in physical and technical match performance variables in football players promoted from the Spanish Second Division to the First Division

## References

[1] Wallace JL, Norton KI. Evolution of WorldCup soccer final games 1966–2010: Game structure, speed and play patterns. J Sci Med Sport. 2014; 17(2):223–8. 23643671 10.1016/j.jsams.2013.03.016

[2] Barnes C, Archer DT, Hogg B, Bush M, Bradley PS. The Evolution of Physical and Technical Performance Parameters in the English Premier League. Int J Sports Med. 2014; 35(13):1095–100. 25009969 10.1055/s-0034-1375695

[3] Bradley P, Archer D, Hogg R, Schuth G, Bush M, Carling C, et al. Tier-Specific Evolution of Match Performance Characteristics in the English Premier League: It’s Getting Tougher at the Top. J Sports Sci. 2015; 34. 10.1080/02640414.2015.1082614 26359805

[4] Bush M, Barnes C, Archer DT, Hogg B, Bradley PS. Evolution of match performance parameters for various playing positions in the English Premier League. Hum Mov Sci. 2015; 39:1–11. 25461429 10.1016/j.humov.2014.10.003

[5] Errekagorri I, Castellano J, Echeazarra I, López-Del Campo R, Resta R. A longitudinal analysis of technicaltactical and physical performance of the teams in the Spanish LaLiga Santander: An eight-season study. Biol Sport. 2022; 39(2):389–96. 35309534 10.5114/biolsport.2022.105331 PMC8919887

[6] Konefał M, Chmura P, Zając T, Chmura J, Kowalczuk E, Andrzejewski M. Evolution of technical activity in various playing positions, in relation to match outcomes in professional soccer. Biol Sport. 2019; 36(2):181–9. 31223196 10.5114/biolsport.2019.83958 PMC6561231

[7] Lago-Peñas C, Lorenzo-Martinez M, López-Del Campo R, Resta R, Rey E. Evolution of physical and technical parameters in the Spanish LaLiga 2012–2019. Sci Med Footb. 2022; 0(0):1–6. 10.1080/24733938.2022.2049980 35243954

[8] Yi Q, Liu H, Nassis GP, Gómez MÁ. Evolutionary Trends of Players’ Technical Characteristics in the UEFA Champions League. Front Psychol. 2020; 11. 10.3389/fpsyg.2020.01032 PMC7308470 32612550

[9] Gómez-Carmona CD, Bastida-Castillo A, Ibáñez SJ, Pino-Ortega J. Accelerometry as a method for external workload monitoring in invasion team sports. A systematic review. PLOS ONE. 2020; 15(8):e0236643. 32841239 10.1371/journal.pone.0236643 PMC7447012

[10] Zhou C, Gómez MÁ, Lorenzo A. The evolution of physical and technical performance parameters in the Chinese Soccer Super League. Biol Sport. 2020; 37(2):139–45. 32508381 10.5114/biolsport.2020.93039 PMC7249799

[11] Sarmento H, Clemente FM, Araújo D, Davids K, McRobert A, Figueiredo A. What Performance Analysts Need to Know About Research Trends in Association Football (2012–2016): A Systematic Review. Sports Med. 2018; 48(4):799–836. 29243038 10.1007/s40279-017-0836-6

[12] Bradley PS, Carling C, Gomez Diaz A, Hood P, Barnes C, Ade J, et al. Match performance and physical capacity of players in the top three competitive standards of English professional soccer. Hum Mov Sci. 2013; 32(4):808–21. 23978417 10.1016/j.humov.2013.06.002

[13] Pons E, Ponce Bordón J, García J, López del Campo R, Resta R, Peirau X, et al. A Longitudinal Exploration of Match Running Performance during a Football Match in the Spanish La Liga: A Four-Season Study. Int J Environ Res Public Health. 2021; 18:1133. 33525322 10.3390/ijerph18031133 PMC7908616

[14] Gomez-Piqueras P, González-Víllora S, Castellano J, Teoldo da Costa I. Relation between the physical demands and success in professional soccer players. J Hum Sport Exerc. 2018; 14.

[15] Castellano J, Casamichana D. What are the differences between first and second divisions of Spanish football teams? Int J Perform Anal Sport. 2015; 15(1):135–46.

[16] Saeterbakken A, Haug V, Fransson D, Grendstad H, Gundersen H, Moe V, et al. Match Running Performance on Three Different Competitive Standards in Norwegian Soccer. Sports Med Int Open. 2019; 03:E82–8. 10.1055/a-0943-3682 PMC6795532 31624770

[17] Morgans R, Adams D, Mullen R, Sacramento J, McLellan C, Williams M, et al. A Comparison of Physical and Technical Match Performance of a Team Competing in the English Championship League and Then the English Premier League Following Promotion. Int J Sports Sci Coach. 2015; 10:543–50.

[18] Oliva-Lozano JM, Fortes V, Krustrup P, Muyor JM. Acceleration and sprint profiles of professional male football players in relation to playing position. PLOS ONE. 2020; 15(8):e0236959. 32760122 10.1371/journal.pone.0236959 PMC7410317

[19] Felipe JL, Garcia-Unanue J, Viejo-Romero D, Navandar A, Sánchez-Sánchez J. Validation of a Video-Based Performance Analysis System (Mediacoach^®^) to Analyze the Physical Demands during Matches in LaLiga. Sensors. enero de 2019; 19(19):4113. 10.3390/s19194113 PMC6806213 31547591

[20] Di Salvo V, Pigozzi F, González-Haro C, Laughlin MS, De Witt JK. Match performance comparison in top English soccer leagues. Int J Sports Med. 2013; 34(6):526–32. 23184481 10.1055/s-0032-1327660

[21] Mitrotasios M, Gonzalez-Rodenas J, Armatas V, Aranda R. The creation of goal scoring opportunities in professional soccer. Tactical differences between Spanish La Liga, English Premier League, German Bundesliga and Italian Serie A. Int J Perform Anal Sport. 2019; 19(3):452–65.

[22] Sarmento H, Pereira A, Matos N, Campanico J, Anguera MT, Leitão J. English Premier League, Spain’s La Liga and Italy’s Serie’s A-What’s Different? Int J Perform Anal Sport. 2013; 13:773–89.

[23] Dalen T, Jørgen I, Gertjan E, Geir Havard H, Ulrik W. Player Load, Acceleration, and Deceleration During Forty-Five Competitive Matches of Elite Soccer. J Strength Cond Res. 2016; 30(2):351–9. 26057190 10.1519/JSC.0000000000001063

[24] Mallo J, Mena E, Nevado F, Paredes V. Physical Demands of Top-Class Soccer Friendly Matches in Relation to a Playing Position Using Global Positioning System Technology. J Hum Kinet. 2015; 47:179–88. 26557202 10.1515/hukin-2015-0073 PMC4633253

[25] Rico-González M, Oliveira R, Palucci Vieira LH, Pino-Ortega J, Clemente FM. Players’ performance during worst-case scenarios in professional soccer matches: a systematic review. Biol Sport. 2022; 39(3):695–713. 35959320 10.5114/biolsport.2022.107022 PMC9331336

[26] Ju W, Doran D, Hawkins R, Evans M, Laws A, Bradley PS. Contextualised high-intensity running profiles of elite football players with reference to general and specialised tactical roles. Biol Sport. 2023; 40(1):291–301. 36636193 10.5114/biolsport.2023.116003 PMC9806759

[27] Yi Q, Jia H, Liu H, Gómez MÁ. Technical demands of different playing positions in the UEFA Champions League. Int J Perform Anal Sport. 2018; 18(6):926–37.

[28] Duarte R, Araújo D, Correia V, Davids K. Sports teams as superorganisms: implications of sociobiological models of behaviour for research and practice in team sports performance analysis. Sports Med Auckl NZ. 2012; 42(8):633–42. 10.2165/11632450-000000000-00000 22715927

