# Supplementary material for: Changes in physical and technical match performance variables in football players promoted from the Spanish Second Division to the First Division
Source: Biol Sport. 2023 Aug 8;41(1):217–25. doi: 10.5114/biolsport.2024.127386 (PMC10765445; doi:10.5114/biolsport.2024.127386)
Supplement: Changes in physical and technical match performance variables in football players promoted from the Spanish Second Division to the First Division [file JBS-41-50670-s1.pdf]

Supplementary materials

Table S1. Comparative analysis of physical performance variables according to the competitive level and its interaction with playing position (mean ± standard deviation).

Variable (unit)	Competitive level		Division			Division*Position		
	Second Division	First Division	F[1]	P[2]	ES[3]	F[1]	P[2]	ES[3]
HIR efforts (n)	30.6±9.1	33.4±10	31.9	<0.001	0.258	1.24	0.298	0.051
HIR distance (m)	526.3±176.1	555.6±188.3	10.8	0.001	0.106	0.36	0.832	0.016
TD (m)	10122.5±840.2	10176.6±867.1	8.05	0.006	0.080	3.95	0.005	0.147

Note: [1]F = F-value [2]P = ANOVA of repeated measures; [3]ES = effect size: partial eta squared

Table S2. Comparative analysis of physical performance variables according to the competitive level and playing positions (mean ± standard deviation).

Variable (unit)	Competitive level			p
	Playing position	Second Division	First Division	
HIR efforts (n)	CD	22.2±1.4	24.4±1.5	0.026
	FB	33.4±1.6	36.6±1.7	0.007
	CM	30.8±1.2	33.2±1.3	0.009
	WM	39.1±2.2	44.9±2.3	<0.001
	FW	37.4±2.4	39.9±2.6	0.147
HIR distance (m)	CD	361.4±26.4	385.2±28.4	0.151
	FB	601.4±30.8	641.4±33.2	0.040
	CM	519.4±23.8	541.9±25.6	0.130
	WM	708.9±40.5	759.7±43.7	0.047
	FW	646.0±44.8	666.0±48.3	0.475
TD (m)	CD	9302.4±116.9	9271.8±127.0	0.709
	FB	10089.2±136.8	10183.3±148.6	0.328
	CM	10835.2±105.4	10767.2±114.5	0.359
	WM	10306.5±179.8	10467.5±195.3	0.203
	FW	9803.5±198.7	10320.7±9891.8	<0.001
